# Associations between individual perceptions of PM_2.5_ pollution and pulmonary function in Chinese middle-aged and elderly residents

**DOI:** 10.1186/s12889-020-08713-6

**Published:** 2020-06-10

**Authors:** Qi Chen, Jiayao Zhang, Yan Xu, Hong Sun, Zhen Ding

**Affiliations:** 1 Jiangsu Provincial Center for Disease Control and Prevention, Jiangsu Road 172, 210009 Nanjing, PR China; 2 Jiangsu Institute of Parasitic Disease, Meiyuan Yang Alley 117, 214064 Wuxi, PR China

**Keywords:** PM_2.5_, Risk perception, Elderly, Policy

## Abstract

**Background:**

PM_2.5_ pollution has become a major public health concern in urban China. Understanding the residents’ individual perceptions toward haze pollution is critical for policymaking and risk communication. However, the perceptions of middle-aged and elderly residents, who particularly vulnerable to haze pollution, are poorly understood. In this study, we aimed to explore their risk perceptions of haze pollution and investigate its relationship with health status and pulmonary function parameters.

**Methods:**

A cross-sectional study of 400 randomly sampled individuals (aged 40 to 90 years) was conducted in Wuxi, a typical PM_2.5_-polluted city in Jiangsu Province, China (during 2015–2017, daily average concentration of PM_2.5_ was 52.7 μg/m^3^). Each participant’s demographic and health information, individual perception and pulmonary function outcomes were collected to explore the relationships between perception factors and personal characteristics and pulmonary function parameters, using linear models.

**Results:**

We found that the mean values for *controllability* (5 ± 2.8) and *dread of risk to oneself* (levels of fear for haze-related harm to oneself) (6.9 ± 2.5) were the lowest and the highest values, respectively, in our study. Education and average family income were positively related with all individual perception factors, while age was negatively associated. A history of respiratory disease was positively associated with all individual perception factors except *controllability*. Significant positive associations were observed between PEF (coefficients ranged from 0.18 to 0.22) and FEF75% (coefficients ranged from 0.18 to 0.29) with a variety of individual perception factors.

**Conclusions:**

There were a lack of concern and knowledge, weak self-protection consciousness and a strong dread of PM_2.5_ pollution among the middle-aged and elderly residents in Wuxi. Their individual perceptions were associated with age, education levels, average family income, history of respiratory disease, PEF and FEF75%. Our findings may help policymakers develop effective policies and communication strategies to mitigate the hazards of haze among older residents.

## Background

Particulate air pollution is a severe global issue. The evidence of a link between increased mortality and particulate matter exposure has been established in more than 600 cities across the globe [1].

With rapid economic growth, energy consumption and air pollutant emissions have substantially increased in China [2]. Fine particulate matter with a diameter of < 2.5 μm (PM_2.5_) pollution has become one of the most severe environmental problems, especially in highly industrialized urban areas of China [3]. Numerous studies have established that PM_2.5_ pollution threatens human health in many ways; it increases the morbidity and mortality of respiratory and cardiovascular diseases, impairs humans’ pulmonary and cognitive function, and has adverse effects on mental health and well-being [4–6]. Although PM_2.5_ is hazardous to the whole population, elderly people are especially vulnerable [7].

In addition to health impacts, large-scale PM_2.5_ pollution in Chinese urban areas have also caused some social problems. In the winter of 2018, many areas of China had haze pollution alerts and closed important expressway stations due to the serious smog [8]. In areas with high concentrations of PM_2.5_, PM_2.5_ pollution-related diseases cause additional medical expenses, work time loss and GDP loss [9, 10].

Because frequent haze pollution has led to a rise in Chinese public concern and has caused the potential risk of social unrest [11]. The Chinese government has thus far established a series of environmental regulations to control industrial and vehicular emissions, encourage clean energy and set up air pollution warning systems and action plans for haze episodes [12–14].

According to the Chinese National Ambient Air Quality Standard, annual mean PM_2.5_ concentration should not exceed 35 μg/m^3^ [15]. However, all the annual mean PM_2.5_ concentrations of the 13 cities in Jiangsu province exceeded this standard during 2015–2017 [16]. Real-time PM_2.5_ concentrations have been made available to the public in recent years. However, some recent studies suggest that less than half Chinese urban residents check the daily air quality index (AQI), their most favored ways to obtain haze-related information were from television, internet and newspapers [11, 17]. These findings indicate that it is important for the government to deliver effective environmental education and risk communication to local residents through their favored ways.

“Risk perception” is how people judge the magnitude and degree of risk with their intuition [18]. It is a very important and effective indicator of public concern about air pollution. Risk perception can guide individuals’ self-protective behaviors and help them respond to government work [19]. On the other hand, understanding the population’s risk perception can help the government engage in efficient risk communication to bridge the gap of risk perception between the experts and the public, and to create effective policies to protect public health and mitigate potential adverse socioeconomic impacts.

Given that a proper understanding of individual perceptions of air pollution is critical for policymaking and risk communication, many studies have been conducted to explore the public’s perception of air pollution in recent years [20–25]. Previous studies have reported that a lower level of education and income might be associated with more dissatisfaction with respect to air pollution [20, 21]. Qian and Kim et al. found that women and younger people are more sensitive to air pollution risks [17, 22]. However, other studies have indicated that middle-aged and elderly people perceived greater risk and health threats associated with air pollution [23–25], and people with higher education and income levels tend to be more concerned about air pollution [26–28]. Moreover, individual perception could be influenced by health status, thermal sensations, and personal experiences with air pollution [19, 29, 30].

The majority of previous studies have focused on a relatively young population. Their respondents were younger and more educated than the average individual in the targeted population, this was potentially because older, uneducated people have fewer chances to participate in such studies and have greater difficulty in understanding questions [19, 30, 31]. Middle-aged and elderly residents are particularly vulnerable to air pollution [32, 33]; therefore, it is crucial to understand their risk perceptions and develop targeted policy strategies to protect them. However, it might be inappropriate to generalize the conclusions of previous studies directly to them. Because compare to young people, middle-aged and elderly individuals are more likely to have cardiovascular diseases and worse pulmonary function [34, 35]. Those characteristics may largely affect their risk perceptions.

In light of these findings, we conducted our study among the middle-aged and elderly residents (age between 40 to 90 years old) from Wuxi, an important economic and industry center in the Yangtze River Delta region. The aims of this study were to explore middle-aged and elderly urban residents’ risk perceptions of haze pollution and to determine the relationship between health status and pulmonary function parameters and risk perception.

## Methods

### Questionnaire

The questionnaire was composed of two parts. The first part of the questionnaire surveyed demographic information and health status. For health status, we collected information about *history of cardiovascular disease* and *history of respiratory disease.* The participants with diagnosed hypertension, arrhythmia, coronary heart disease, myocardial infarction or any other cardiovascular disease that had been reported to be related to PM_2.5_ pollution were defined as “have a history of cardiovascular disease”. Those with diagnosed asthma, chronic obstructive pulmonary disease, lung cancer, chronic respiratory inflammation or acute respiratory inflammation (occurring within 1 year) were defined as “have a history of respiratory disease”.

The second part consisted of eight questions that reflected individual perceptions of PM_2.5_ pollution and its related health effects. It was designed based on the psychometric method and adapted from a previous study by Ban et al. [19]. The eight perception factors were *concern*, *severity of air pollution*, *severity of health effects*, *knowledge*, *familiarity*, *dread of risk to oneself*, *dread of risk to others* and *controllability*. Each question asked the participants to give a score from 1 to 10 to reflect their perception levels (Specific questions and risk characteristics’ definitions are shown in Additional file 1). In this study, the total Cronbach’s alpha value was 0.88.

### Sample selection

This study was conducted in Wuxi, an important industry and economy center in Yangtze River Delta region. According to the government monitoring data, the mean concentration of PM_2.5_ in Wuxi was 52.7 μg/m^3^ between 2015 and 2017, which was 5.3 times higher compare to the WHO air quality guideline’s stipulation (PM_2.5_ should not exceed 10 *μg*/*m*^*3*^ annual mean) [16].

Considering the large population (more than 6 million) of Wuxi, we used a two-step sampling method in this study to narrow down the sampling population and capture key population characteristics. First, one district and one county were randomly selected from 5 districts and 2 counties, respectively, in the city. Then, based on probabilities proportional to population size, all communities in the district/county with more than 10,000 residents selected as our basic sample units. In the second step, one such community was randomly selected from both the chosen district and county, and 200 middle-aged and elderly residents (aged 40 to 90 years, living at their current residence for more than 3 years before this study) from each community were randomly selected by turns. We built a table contains the IDs of all the residents who met the criteria, and then the first resident was chosen by generating a random number; the other 199 residents were in turn chosen by equal interval numbers based on the previous resident ID. If the selected resident refused to participate our survey, another resident with the next ID number was chosen, until we successfully interviewed 200 residents in each area. Overall, 416 residents were approached, of whom 400 responded (response rate: 96.1%).

Formula for estimating sample size is as follows: N = $\frac{U_{\alpha}^2{\sigma}^2}{\varDelta^2}$, where take α = 0.05 as significance level, σ is the standard deviation, σ = 2.5, Δ = $\frac{1}{10}\sigma$ =0.25,

$$ \mathrm{N}=\frac{1.96^2{2.5}^2}{0.25^2}=384.16=384 $$

Power calculation was performed using PASS15.0 software for our sample size. In this study, the estimated correlation coefficients between pulmonary function outcomes and individual perception of PM2.5 were more than 0.2. We set the significance level at α = 0.05, the estimated power for the sample size of 400 was more than 98%.

All participants were interviewed face-to-face by a member of our research team, and the pulmonary function test was performed by trained specialists. A total of 400 residents from the two communities completed the questionnaire, 398 of which successfully underwent pulmonary function testing.

### Pulmonary function test

Pulmonary function tests were conducted by trained specialists using a portable spirometer (MINATO™ AS-507, Japan), in accordance with the guidelines provided by the American Thoracic Society/European Respiratory Society (ATS/ERS). Pulmonary function parameters, including FVC (forced vital capacity, L), FEV_1_ (forced expiratory volume in the first second, L), PEF (peak expiratory flow, L/s), FEF_25%_ (forced expiratory flow at 25% of forced vital capacity, L/s) and FEF_75%_ (forced expiratory flow at 75% of forced vital capacity, L/s), were selected for statistical analysis.

### Statistical models

Descriptive statistics were used to illustrate the sample characteristics, pulmonary function outcomes and individual perceptions of PM2.5 pollution.

Results of sample characteristics were expressed as numbers and rates, while pulmonary function outcomes and individual perceptions of PM_2.5_ pollution were expressed as mean and standard deviation (SD). Student’s t-test was used to compare the individual perception factors of PM_2.5_ pollution between the middle-aged and elderly groups.

To examine the effects of demographic and health status variables for individual perceptions of PM_2.5_ pollution, we constructed linear regression equation models. The model included each individual perception factor as a dependent variable. Age was divided into five groups: 41–50 years old = 1, 51–60 years old = 2, 61–70 years old = 3, 71–80 years old = 4, and 81–90 years old = 5. Education was divided into four groups: primary school and below = 1, middle school = 2, high school = 3, and college and above = 4. Income was divided into three categories (CNY/year): < 20,000 = 1, 20,000–35,000 = 2, and > 35,000 = 3. Other binary variables included gender (male = 1, female = 2), history of cardiovascular disease (no = 0, yes = 1), and history of respiratory disease (no = 0, yes = 1).

Generalized linear models (GLMs) were used to explore the relationship between pulmonary function parameters and individual perception factors of PM_2.5_. Considering pulmonary function (spirometric measures FEV1, FVC., etc.) accelerates the loss in function after 65 years of age, and age was reported as an important influencing factor of risk perception in previous studies [36]. We conducted separate analyses for the middle-aged group (aged 41–65, *N* = 195) and the elderly group (aged 66–90, *N* = 203). Age in years, education, average family income and history of respiratory disease were included in all initial models. Since some of these variables were correlated, we used stepwise regression to remove highly correlated covariates from the initial models. The variance inflation factor (VIF) of the models after stepwise regression selection were all < 5.

All statistical analyses were performed using R software (version 3.3.1, R Foundation for Statistical Computing, http://cran.r-project.org/). Generalized linear models (GLMs) were fitted using the *splines* package. *P*-values < 0.05 were considered significant.

## Results

A total of 416 residents were approached, of whom 400 responded (response rate: 96.1%, completion rate: 100%). Among the responded residents, 398 of these successfully underwent pulmonary function tests. The demographic characteristics and health status of the participants are summarized in Table 1. There were 49.8% females and 51.2% participants aged over 65 years. The majority of the participants’ education levels were below high school (70.8%), and only 30% participants had an average family income of over 35,000 CNY/year. Very few of the participants had ever used a household air purifier (4.8%). A total of 46% of the participants had a history of cardiovascular disease, and 17% had a history of respiratory disease.

**Table 1:** Sample description

Characteristic	Groups	Distribution (n, %)
Age (years old)	41–50	67 (16.8%)
	51–60	79 (19.8%)
	61–70	88 (22%)
	71–80	81 (20.2%)
	81–90	85 (21.2%)
Gender	Male	201 (50.2%)
	Female	199 (49.8%)
Education	Primary school and below	139 (34.8%)
	Middle school	144 (36%)
	High school	80 (20%)
	College and above	37 (9.2%)
Average family income (CNY/year)	< 20,000	136 (34%)
	20,000–35,000	144 (36%)
	> 35,000	120 (30%)
Household air purifier use	Yes	19 (4.8%)
	No	381 (95.2%)
History of cardiovascular disease	Yes	184 (46%)
	No	216 (54%)
History of respiratory disease	Yes	68 (17%)
	No	332 (83%)

### Pulmonary function outcomes

Descriptive statistics of the participants’ pulmonary function outcomes are provided in Additional file 2. According to previous studies, pulmonary function (spirometric measures FEV1, FVC., etc.) declines with increasing age, and the loss in function will especially accelerate after 65 years of age [36, 37]. Therefore, we calculated the pulmonary function outcomes of the participants aged 41–65 (middle-aged group) and of those aged 66–90 (elderly group) separately. The analysis showed that the pulmonary function outcomes of the middle-aged group were all significantly higher than those of the elderly group.

### Individual perceptions

**Fig. 1:**
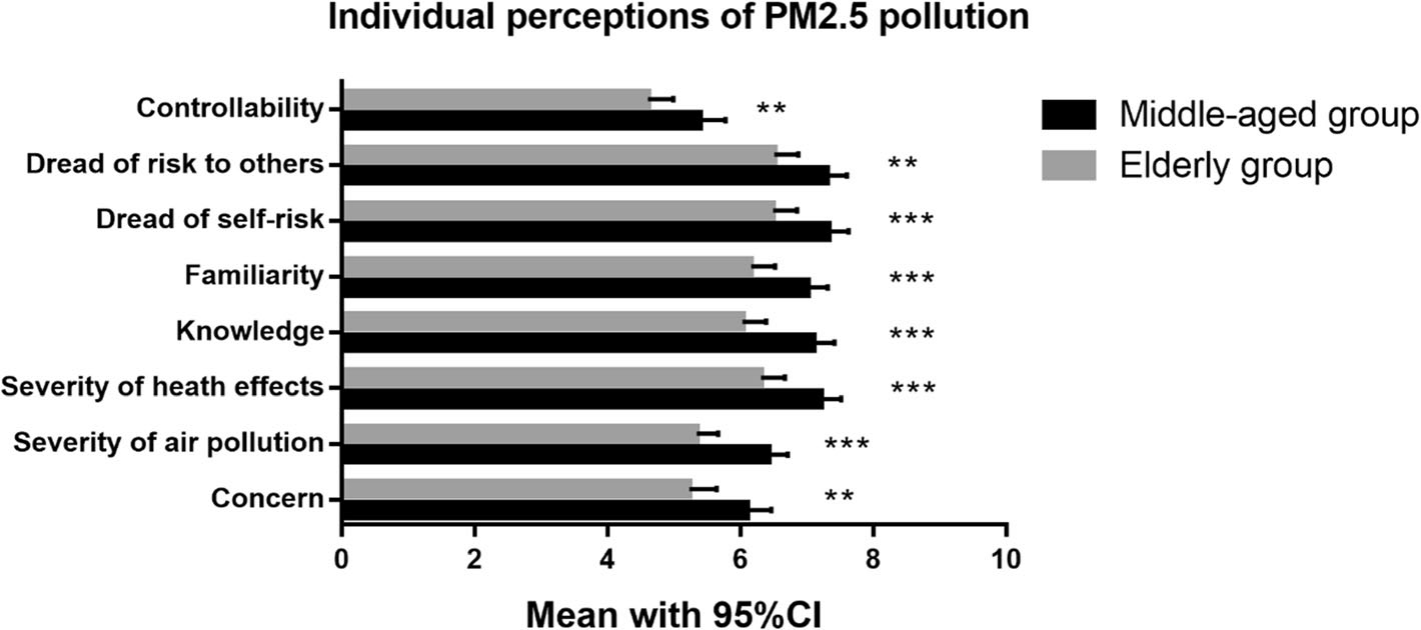
Comparisons of individual perception factors of PM_2.5_ pollution between the middle-aged and elderly groups (**P < 0.01, ***P < 0.001)

As seen in Table 2, the mean values of individual perception factors in the total sample of participants ranged from 5 to 6.9. *Dread of risk to oneself* scored highest, and *controllability* scored lowest in our study. In addition, we compared the differences in individual perception between the middle-aged group and the elderly group through an independent-sample *t*-test analysis. We found that the values of the individual perception factors of the middle-aged group were all significantly higher than those of the elderly group (indicated in Fig. 1).

**Table 2:** Individual perceptions of PM_2.5_ pollution (mean ± SD)

Perception factors	Total (*N* = 400)	Age: 41–65 (*N* = 195)	Age: 66–90 (*N* = 205)
Concern	5.7 ± 2.8	6.1 ± 2.6	5.2 ± 3
Severity of air pollution	5.9 ± 2.3	6.4 ± 2.1	5.3 ± 2.4
Severity of health effects	6.8 ± 2.4	7.2 ± 2.2	6.3 ± 2.6
Knowledge	6.6 ± 2.5	7.1 ± 2.2	6 ± 2.6
Familiarity	6.6 ± 2.4	7 ± 2.2	6.2 ± 2.6
Dread of risk to oneself	6.9 ± 2.5	7.3 ± 2.2	6.5 ± 2.6
Dread of risk to others	6.9 ± 2.4	7.3 ± 2.1	6.5 ± 2.6
Controllability	5 ± 2.8	5.4 ± 2.7	4.6 ± 2.8

### Factors influencing individual perceptions of PM_2.5_

To reveal the influencing factors, we employed regression models to analyze the effects of demographic and health status variables on individual perceptions of PM_2.5_ pollution (Table 3). We found that *education* (coefficients ranging from 0.45 to 0.94) and *average family income* (coefficients ranging from 0.39 to 0.72) were positively associated with all individual perception factors; in addition, *age* (coefficients ranged from − 0.41 to − 0.28) was negatively associated with all individual perception factors. *History of respiratory disease* (coefficients ranging from 0.84 to 1.47) was positively associated with all individual perception factors except *controllability*. No significant correlation was found between *history of cardiovascular disease* and *gender* and any individual perception factor of PM_2.5_.

**Table 3:** Regression analysis of influencing factors on individual perception of PM_2.5_ among middle-aged and elderly residents (*N* = 398)^a^

	Concern	Severity of air pollution	Severity of health effects	Knowledge	Familiarity	Dread of risk to oneself	Dread of risk to others	Controllability
Age	−0.28**	−0.4**	− 0.35**	−0.41**	− 0.32**	−0.37**	− 0.35**	− 0.31**
Gender	− 0.35	0.09	− 0.36	− 0.4	− 0.24	−0.27	− 0.29	0.08
Education	0.9**	0.54**	0.85**	0.93**	0.86**	0.9**	0.94**	0.45**
Average family income	0.53**	0.39**	0.72**	0.48**	0.55**	0.59**	0.63**	0.39*
History of cardiovascular disease	−0.42	−0.01	0.03	−0.27	−0.18	− 0.02	0.07	− 0.46
History of respiratory disease	0.84*	1.06**	1.33**	1.09**	1.17**	1.47**	1.4**	0.03

^a^: **P < 0.01, *P < 0.05

### Associations between pulmonary function outcomes and individual perception of PM_2.5_

We analyzed the associations between pulmonary function outcomes and individual perceptions, with separate analyses conducted for the middle-aged group (aged 41–65, *N* = 195) and the elderly group (aged 66–90, *N* = 203). Age in years, education, average family income and history of respiratory disease were included in all initial models, and stepwise regression was used to remove variables.

As shown in Table 4, we observed significant positive associations of PEF with *dread of risk to oneself* (coefficient = 0.2, *P* < 0.05) and *dread of risk to others* (coefficient = 0.22, *P* < 0.05), and we observed positive associations of FEF_75%_ with *severity of health effects* (coefficient = 0.2, *P* < 0.05), *familiarity* (coefficient = 0.18, *P* < 0.05), *dread of risk to oneself* (coefficient = 0.24, *P* < 0.01) *and dread of risk to others* (coefficient = 0.26, *P* < 0.01) among the middle-aged residents. No significant correlation was found between other pulmonary function indexes and individual perception factors.

**Table 4:** Regression analysis of pulmonary function outcomes on individual perception of PM_2.5_ among residents aged 41–65 (*N* = 195)^a^

	Severity of air pollution	Severity of health effects	Knowledge	Familiarity	Dread of risk to oneself	Dread of risk to others
PEF, L/s	0.18*	–	0.14	0.16	0.2*	0.22*
FEF_25%_, L/s	–	−0.22	–	−0.23	−0.2	− 0.17
FEF_75%_, L/s	0.14	0.2*	0.16	0.18*	0.24**	0.26**

^a^: **P < 0.01, *P < 0.05

-: removed by stepwise regression selection

All initial models included age, education, average family income and history of respiratory disease, and then underwent stepwise regression selection

Similar associations were observed among the elderly residents (Table 5). We observed significant positive associations of FEF_75%_ with *knowledge* (coefficient = 0.25, *P* < 0.05), *familiarity* (coefficient = 0.28, *P* < 0.05), *dread of risk to oneself* (coefficient = 0.29, *P* < 0.05) *and dread of risk to others* (coefficient = 0.23, *P* < 0.05). Furthermore, we also found that FEV_1_ and FVC were positively associated with *familiarity.*

**Table 5:** Regression analysis of pulmonary function outcomes on individual perception of PM_2.5_ among residents aged 66–90 (*N* = 203) ^a^

	Severity of air pollution	Severity of health effects	Knowledge	Familiarity	Dread of risk to oneself	Dread of risk to others
FEV_1_, L	–	–	–	0.65*	–	–
FVC, L	–	–	–	0.55*	–	–
FEF_75%_, L/s	–	0.21	0.25*	0.28*	0.29*	0.23*

^a^: *P < 0.05

-: removed by stepwise regression selection

All initial models included age, education, average family income and history of respiratory disease, and then underwent stepwise regression selection

## Discussion

In this study, we explored middle-aged and elderly urban residents’ risk perception of haze pollution and found they were associated with their health status and pulmonary function parameters.

Our study found that the mean values for self-reported *controllability* and *concern* were the lowest, while the mean values for *dread* (*dread of risk to oneself* and *dread of risk to others*) were the highest in our study. These results are consistent with those of previous studies. For example, Liu et al. reported that only 42.5% of the respondents in Shanghai, Wuhan and Nanchang paid attention to air pollution-related indicators, and Lan et al. reported that only 14.6% of respondents in Nanchang checked the air quality index regularly [11, 38]. Meanwhile, 78.8% of the respondents in Ningbo felt dread toward the possible aggravation of the haze, and 83% of respondents in Nanchang worried about the potential adverse impact on their respiratory system caused by a high level of air pollution [17, 38]. Despite the dread of haze pollution, less than 5% participants in our study had ever used air purifiers to improve indoor air quality; this ratio is much lower than that reported among younger residents in another study conducted in Nanjing (15.2% air purifier use) [19]. This may be due to the low levels of self-perceived *controllability* in our study; in other words, the residents do not believe that they can effectively reduce the health risks associated with haze by engaging in self-protective behaviors.

Previous studies have indicated that individual perceptions of air pollution could be influenced by many factors, such as age [39, 40], gender [41], education level [42], family income [43], individual experiences [19], and health symptoms [30]. Supporting the previous findings, we found that *education* and *average family income* were positively associated and *age* was negatively associated with all individual perception factors. *History of respiratory disease* was positively associated with all individual perception factors except *controllability* in our study. However, although cardiovascular injury is one of the most important health hazards of air pollution, no significant correlation was found between *history of cardiovascular disease* and any individual perception factor of PM_2.5_. This result indicates that the residents with cardiovascular disease may not identify haze pollution as a health threat to their disease and did not pay more attention to it than the healthy group did. A good knowledge of health risks associated with haze will help promote self-protective behaviors [19]. Therefore, it is important to provide education about haze-related health risks and self-protective behaviors among residents with cardiovascular diseases. A similar situation was found by Liu et al., in which only 21.2% of the respondents considered heart problems a health consequence of air pollution [11]. Nevertheless, as indicated by the self-reported perceived *knowledge* level (mean score: 6.6 ± 2.5), Wuxi’s middle-aged and elderly residents believed that they have adequate haze-related knowledge. Taken together, these results may reveal a potential obstacle in current air pollution-related health education: there is a gap between residents’ self-perceived knowledge level and their actual level, which may cause insufficient self-protective behavior among vulnerable groups and incorrect risk perceptions. Therefore, we suggest that government managers develop targeted health education strategies and risk communication messages for vulnerable groups, especially for residents with cardiovascular diseases.

Both pulmonary function outcomes and individual perceptions could be influenced by age, and they were significantly different between the middle-aged and elderly groups in this study (shown in Additional file 2 and Fig. 1). Therefore, we analyzed the associations between pulmonary function outcomes and individual perceptions separately among the middle-aged group (aged 41–65, *N* = 195) and the elderly group (aged 66–90, *N* = 203). It is very interesting that we observed better pulmonary function outcomes were related to higher self-perceived levels of *severity of health effects*, *knowledge*, *familiarity, dread of risk to oneself* and *dread of risk to others* in both middle-aged and elderly groups*.* These results indicated that residents with worse pulmonary function might lack knowledge of the hazards of PM_2.5_ pollution and did not consider PM_2.5_ pollution as a severe health threaten. As reported in a previous study, lower knowledge and dread levels may result in less self-protection behaviors during haze pollution [19], and less self-protection may further worsen pulmonary function. These results emphasized the importance of environmental education and risk communication among the residents with poor pulmonary function.

In this study, we found that FEF_75%_ was associated with *familiarity*, *dread of risk to oneself and dread of risk to others* in both middle-aged and elderly groups. These results indicated that FEF_75%_ might be used as an indicator for hospital-based health education to identify who may need to improve their knowledge and who may need to relieve their anxiety toward the potential health risks caused by PM_2.5_. Our findings suggested that policymakers should consider the health status of target population when making health education and risk communication strategies.

To the best of our knowledge, this is the first study specifically focused on risk perception among Chinese middle-aged and elderly residents, who are generally defined as the vulnerable group to PM_2.5_ pollution. We identified the associations between residents’ individual risk perceptions with their health status and pulmonary function outcomes. We discussed several policy implications in the sections of discussion in this article, our results may be helpful for policymakers to make more effective policies.

Our study also has some limitations. First, as a cross-sectional study, the pulmonary function tests were performed on the same day as the questionnaires, and we observed that the residents’ pulmonary function outcomes were associated with their individual perceptions. As a previous study reported that higher levels of risk perceptions may promote residents take additional protective actions [19]. In this study, we cannot decide whether higher levels of risk perceptions have helped protect residents’ pulmonary function. Second, our study participants were sampled based on communities, those with severe respiratory and cardiovascular disease may not sufficiently included in our survey. Moreover, all the questionnaires were collected during the winter of 2018, season might also influence the residents’ risk perceptions. In future work, we plan to conduct surveys in different seasons and include more participants from both communities and hospitals.

Despite these limitations, our study provides a reference for government managers about the associations between Chinese middle-aged and elderly urban residents’ individual risk perceptions with their health status and pulmonary function outcomes for the first time, and lays the foundation for subsequent researchers.

## Conclusions

Our study suggests that middle-aged and elderly residents may lack concern about air pollution, and their perception of *knowledge* might be highly different from their real knowledge. Moreover, they may have no confidence in mitigating PM_2.5_-related health risks by self-protective behaviors, and dread of the hazards of PM_2.5_. Their individual perceptions were associated with age, education levels, average family income, history of respiratory disease and their pulmonary function outcomes. Our findings may help government managers ascertain sensitive groups and make targeted strategies to conduct correct environmental education, strengthen risk communication, relieve dread feelings and encourage self-protective behaviors among middle-aged and elderly residents. Further studies involve more participants from different regions of China to better understand why Chinse residents are not taking sufficient self-protective behaviors during haze days are needed.

## Supplementary information

**Additional file 1: Supplemental Table S1.** Question design and definition of risk characteristics. ^a^ Scale ranges from 1 = Not concerned at all to 10 = Very concerned. ^b^ Scale ranges from 1 = Not serious at all to 10 = Very serious. ^c^ Scale ranges from 1 = Unknown to 10 = High level of knowledge. ^d^ Scale ranges from 1 = Not familiar at all to 10 = Very familiar. ^e^ Scale ranges from 1 = Not serious at all to 10 = Very serious. ^f^ Scale ranges from 1 = No dread at all to 10 = Complete dread. ^g^ Scale ranges from 1 = No dread at all to 10 = Complete dread. ^h^ Scale ranges from 1 = Not controllable at all to 10 = Completely controllable.

**Additional file 2: Supplemental Table S2.** Pulmonary function outcomes (mean ± SD).

## Data Availability

The data that support the findings of this study are available from Jiangsu Provincial Commission of Health and Family Planning; however, restrictions apply regarding the availability of these data, which were used under a license for the current study, and the data are not publicly available. However, data are available from the authors upon reasonable request and with permission of the Jiangsu Provincial Commission of Health and Family Planning. Please contact the corresponding author: Zhen Ding, cqjscdc@126.com.

## Abbreviations

PM_2.5_: Fine particulate matter with a diameter of < 2.5 μm
PPS: Probabilities proportional to population size
SRS: Simple random sampling method
FVC: Forced vital capacity
FEV_1_: Forced expiratory volume in the first second
PEF: Peak expiratory flow
FEF_25%_: Forced expiratory flow at 25% of forced vital capacity
FEF_75%_: Forced expiratory flow at 75% of forced vital capacity
GLMs: Generalized linear models
