# Supplementary material for: Associations between individual perceptions of PM2.5 pollution and pulmonary function in Chinese middle-aged and elderly residents
Source: BMC Public Health. 2020 Jun 10;20:899. doi: 10.1186/s12889-020-08713-6 (PMC7288539; doi:10.1186/s12889-020-08713-6)
Supplement: Supplementary file 1 — Additional file 1: Supplemental Table S1. Question design and definition of risk characteristics. a Scale ranges from 1 = Not concerned at all to 10 = Very concerned. b Scale ranges from 1 = Not serious at all to 10 = Very serious. c Scale ranges from 1 = Unknown to 10 = High level of knowledge. d Scale ranges from 1 = Not familiar at all to 10 = Very familiar. e Scale ranges from 1 = Not serious at all to 10 = Very serious. f Scale ranges from 1 = No dread at all to 10 = Complete dread. g Scale ranges from 1 = No dread at all to 10 = Complete dread. h Scale ranges from 1 = Not controllable at all to 10 = Completely controllable. [file 12889_2020_8713_MOESM1_ESM.docx]

**Additional file 1**

**Supplemental Table 1** Question design and definition of risk characteristics

| Questions | Perception factor | Value |
| --- | --- | --- |
| 1. In your opinion, are you concerned about the haze related information? | Concern | 1-10^a^ |
| 2. In your opinion, is local haze serious? | Severity of air pollution | 1-10^b^ |
| 3. In your opinion, how aware are you of the risk associated with haze? | Perceived knowledge | 1-10^c^ |
| 4. In your opinion, is the risk associated with haze a familiar risk or an unfamiliar risk? | Familiarity | 1-10^d^ |
| 5. In your opinion, is the health risk associated with haze serious? | Severity of health effects | 1-10^e^ |
| 6. In your opinion, if the pollution happens, how do you fear the risk associated with haze to yourself? | Dread of risk to oneself | 1-10^f^ |
| 7. In your opinion, if the pollution happens, how do you fear the risk associated with haze to other local residents? | Dread of risk to others | 1-10^g^ |
| 8. In your opinion, to what degree can you avoid the risk associated with haze by self-protection (such as wearing masks, using air purifiers)? | Controllability | 1-10^h^ |

^a^ Scale ranges from 1 = Not concerned at all to 10 = Very concerned.

^b^ Scale ranges from 1 = Not serious at all to 10 = Very serious.

^c^ Scale ranges from 1 = Unknown to 10 = High level of knowledge.

^d^ Scale ranges from 1 = Not familiar at all to 10 = Very familiar.

^e^ Scale ranges from 1 = Not serious at all to 10 = Very serious.

^f^ Scale ranges from 1 = No dread at all to 10 = Complete dread.

^g^ Scale ranges from 1 = No dread at all to 10 = Complete dread.

^h^ Scale ranges from 1 = Not controllable at all to 10 = Completely controllable
