# Supplementary material for: Associations between individual perceptions of PM2.5 pollution and pulmonary function in Chinese middle-aged and elderly residents
Source: BMC Public Health. 2020 Jun 10;20:899. doi: 10.1186/s12889-020-08713-6 (PMC7288539; doi:10.1186/s12889-020-08713-6)
Supplement: Supplementary file 2 — Additional file 2: Supplemental Table S2. Pulmonary function outcomes (mean ± SD). [file 12889_2020_8713_MOESM2_ESM.docx]

**Additional file 2**

**Supplemental Table 2** Pulmonary function outcomes (mean ± SD)

| Index | Total  (N=398) | Age: 41-65  (N=195) | Age: 66-90  (N=203) | P value |
| --- | --- | --- | --- | --- |
| FEV_1_, L | 1.8 ± 0.7 | 2.1 ± 1.5 | 1.4 ± 2 | <0.001 |
| FVC, L | 2 ± 0.8 | 2.3 ± 0.7 | 1.7 ± 0.7 | <0.001 |
| PEF, L/s | 3.4 ± 1.9 | 4.1 ± 1.9 | 2.8 ± 1.6 | <0.001 |
| FEF_25%_, L/s | 2 ± 1.3 | 2.4 ± 1.4 | 1.7 ± 1.2 | <0.001 |
| FEF_75%_, L/s | 2.4 ± 1.9 | 2 ± 2 | 1.9 ± 1.6 | <0.001 |
